# Rosacea Meibomian Gland Dysfunction Posterior Blepharitis May Be a Marker for Earlier Associated Dyslipidaemia and Inflammation Detection and Treatment with Statins

**DOI:** 10.3390/metabo13070811

**Published:** 2023-06-30

**Authors:** Kenneth G. Ooi, Stephanie L. Watson

**Affiliations:** 1 Corneal Research Group, Discipline of Ophthalmology, Save Sight Institute, Sydney Eye Hospital Campus, Sydney, NSW 2000, Australia; 2 School of Medical Sciences, University of New South Wales, Sydney, NSW 2052, Australia

**Keywords:** rosacea, ocular, meibomian gland dysfunction, blepharitis, cholesterol, hypercholesterolaemia

## Abstract

Posterior blepharitis and dry eye are common disorders with meibomian gland dysfunction (MGD), a principal driver of their pathophysiology. Meibomian gland dysfunction is increasingly prevalent in older populations with contributory hormonal imbalances. The abnormal meibum in MGD has been documented to have an excess of cholesterol with a resultant disruption of the lipid layer of the tear film. This leads to tear film instability due to the inadequate trapping of the aqueous portion of the tear film with resultant evaporative dry eye. Significant morbidity may follow MGD with ocular surface inflammation disrupting both social and work function. Rosacea is a common chronic inflammatory condition of the central face but can have ocular and systemic inflammatory associations. It is especially prevalent in North European populations and can have onset at any age, but commonly presents between thirty and fifty years of age. In ocular rosacea, MGD is a recognised manifestation as is dyslipidaemia. Ocular rosacea can predate cutaneous disease. As yet, there is no directly reported evidence of the efficacy of the early identification and treatment of ocular rosacea with associated dyslipidaemia and systemic inflammation. We posit that MGD in ocular rosacea sufferers may be a marker for dysregulated cholesterol synthesis and inflammation, and that statins maybe a potential therapy. This article introduces potential strategies to utilise ocular rosacea MGD as a possible marker for ophthalmologists, cardiologists, and primary healthcare physicians to treat rosacea-associated dyslipidaemia and systemic inflammation. This could aid in overall cardiovascular morbidity and mortality control for rosacea sufferers, potentially at an earlier age, while also addressing their tear film de-stabilisation through cholesterol lowering and inflammation reduction.

## 1. Introduction

Dry eye, the most common eye disorder [1], is frequently accompanied by blepharitis (eyelid inflammation) which has an overall prevalence of nearly 40%, ranging from around 20% in 10–19 year-olds to 70% in those over 60 [2]. Meibomian gland dysfunction (MGD) is the leading cause of evaporative dry eye and is a type of posterior blepharitis that encompasses inflammation posterior to the grey line of the lid margin [3]. Rosacea is a chronic inflammatory skin disease and is also a common disorder with estimates of upwards of 10% of individuals affected [4,5]. Ocular rosacea is present in up to 58% of cases and is frequently under-recognised by ophthalmologists and other eyecare professionals as ocular signs precede skin involvement in 15% of the cases [6,7,8]. It is characterised most commonly by MGD and posterior blepharitis [9]. Studies have found significant differences in MG secretion (meibum) composition between those with MGD and controls, including the consistent presence of cholesterol esters in MGD patients [10]. Young and middle-aged MGD sufferers, as well as rosacea patients, have both been documented to have a significantly higher blood cholesterol than controls [11,12]. We hypothesise that ocular rosacea patients may have associated elevated cholesterol levels and increased systemic inflammation, such that ocular rosacea maybe a marker that healthcare professionals could use to enable the earlier identification and control of cardiovascular disease and tear film stability through timely cholesterol-lowering and inflammation reduction with statins.

## 2. Rosacea

Rosacea is a chronic inflammatory dermatosis mainly affecting the cheeks, nose, chin, and forehead. It is characterised by recurrent episodes of transient erythema and/or persistent erythema, phymatous changes, papules, pustules, and telangiectasia. Due to facial involvement, it can have a profound impact on quality of life [13]. Men and women have more recently been found to be equally affected, and although reported less frequently in those with skin of colour compared to those with paler skin colours, there may be an under-reporting due to difficulty distinguishing erythema against a darker background [4].

Currently, pathophysiologic modelling suggests a genetic susceptibility and an upregulated and dysregulated innate immune system prone to excessive inflammation and vasodilation, coupled with neurogenic dysregulation from extrinsic triggers [5]. This chronic inflammatory environment is similar to that seen in other immune systemic disorders associated with high C-reactive protein (CRP) levels such as rheumatoid arthritis and the skin disease psoriasis, as discussed by Duman et al. [12]. Inflammation is involved in the genesis of atherosclerosis, and thus cardiovascular disease, as it may lead to structural changes in lipoproteins, thereby negatively affecting cholesterol removal [12,14]. Studies have been reviewed to confirm that the activation of nucleotide binding oligomerisation domain-like receptor 3 may cause IL-1β liberation-inducing alterations in LDL and HDL, thus impeding their ability to breakdown and transport cholesterol. Chronic inflammation therefore in rosacea may be one possible explanation for the associated dyslipidaemia.

Duman et al. reported high total cholesterol, low-density lipoproteins (LDLs) and CRP levels, and a family history of premature cardiovascular disease as being significantly more common in their cohort of rosacea patients than controls [12]. Similarly, Hua et al. reported rosacea patients as being more likely to have dyslipidaemia and hypertension and are at a greater risk of coronary artery disease after adjustment for cardiovascular disease risk factors. When stratified by the onset of age, in those younger than 41 years at onset of rosacea, there was a significant association with dyslipidaemia only. In patients with the onset age of 41 to 55 years, higher risks of dyslipidaemia and coronary artery disease were observed. In patients with onset age older than 55 years, there were significant associations with hypertension, dyslipidaemia, coronary artery disease, and peripheral arterial occlusive disease. Men had higher risks for all cardiovascular disease risk factors and cardiovascular disease than women [14]. 

## 3. Ocular Rosacea

Ocular rosacea is usually mild, although corneal involvement may occur in 41% of cases. Ocular manifestations are usually seen in both eyes, but unilateral or sequential changes can occur [7]. The eyelid margin is characteristically erythematous with telangiectasia, MGD, and, most commonly, posterior blepharitis [9]. In rosacea, meibomian gland hypersecretion occurs with a turbid consistency to the meibum leading to the plugging of the MG and chalazion formation. Ocular rosacea also causes significant meibomian gland loss that can objectively be demonstrated with meibography [15,16]. This also culminates in an abnormal tear film, with a soapy, inferior tear meniscus, leading to evaporative dry eye. Anterior blepharitis is also common and Demodex mites may be seen on the lashes [7].

Chronic inflammation associated with rosacea can lead to a non-specific, chronic conjunctivitis, as well as inferior conjunctival scarring and cicatrisation, granulomas, and phlyctenulosis. Corneal changes can vary from mild inferior dryness to marginal keratitis, corneal vascularisation, lipid keratopathy, scarring, ulceration, and corneal perforation in extreme cases [7,8]. Other corneal pathologies include recurrent epithelial erosions, Salzmann nodules, and phlyctenules [7].

## 4. Meibomian Gland Dysfunction

Meibomian gland dysfunction is a chronic diffuse abnormality of the MG, commonly characterised by terminal duct obstruction and/or qualitative/quantitative changes in the glandular secretions [3]. The obstruction occurs as a result of the hyperkeratinisation of the ductal epithelium and an increased viscosity of the meibum, and can lead to gland dropout, atrophy, and an eventual decreased secretion. This obstructive process can result from numerous factors, both endogenous and exogenous, including age, sex, hormone levels, as well as medications [17]. An impaired delivery of tear film lipids to the ocular surface can cause tear film instability, evaporative dry eye disease, tear hyperosmolarity, and ocular surface inflammation [17,18].

Increased free cholesterol as well as cholesterol esters in the MG may play a role in the pathogenesis of MGD. A higher cholesterol concentration may increase the melting point of meibum, thereby potentially increasing its viscosity with resultant gland orifice plugging [19,20]. Indeed, in a study that included similar numbers of males and females ranging from 27 to 82 years, patients with moderate-to-severe MGD were found to have a higher incidence of dyslipidaemia with respect to elevated total cholesterol and high-density lipoprotein (HDL) than the general population [21]. Subsequently, it has been reported that young and middle-aged MGD patients (mean age 37.6 ± 10.9 years; 23 men and 37 women) with no history of hypercholesterolaemia may have higher blood total cholesterol, LDL, and HDL levels than the controls of equivalent age without MGD [11]. Further confirmation of the latter findings was reported by Braich et al. in a recent study of MGD patients (aged between 20 and 72 years with similar numbers of males and females) versus controls. Additionally, the authors reported that being a male and elderly moderately increased the odds of having dyslipidaemia [22].

The 3-hydroxy-3-methyl-glutaryl-coenzyme A reductase (HMG-CoA reductase) enzyme has been found in the MG as well as the pilosebaceous units and glands of Zeis of human eyelids [23]. Oral statins (HMG-CoA reductase inhibitors) at conventional doses were investigated for an effect on dry eye but were not associated with dry eye symptom reduction in the blue mountains eye study population [24]. Studies have shown an association between statin usage and the presence of moderate-to-severe dry eye symptoms and increased odds of having a dry eye diagnosis [24,25]. An increased meibomian gland atrophy and the deterioration of meibum quality has been shown to progress over 12 months in a prospective study of 98 MGD sufferers on conventional statin doses [26]. More recently, however, a large population-based retrospective study over 14 years of 67,014 patients who used statins as compared to 268,056 patients who did not, found that the incidence of blepharitis was 3.04% in those on statin compared to 3.72% in those not on statins (*p* < 0.001). Patients who used statins had a lower risk of developing blepharitis (adjusted hazard ratio: 0.746, *p* < 0.001) than those who did not. Moreover, the use of a higher intensity statin has been associated with a lower MGD in 535 patients with moderate-to-severe dry eye disease [27]. Topical Atorvastatin to the ocular surface has also been shown to reduce the symptoms and signs of blepharitis-associated dry eye [28]. This may be due to their proposed tear film stabilisation through their MG cholesterol-lowering and pleiotropic anti-inflammatory effects, including the reduction in the pro-inflammatory cytokines, interleukin (IL)-1β, IL-6, IL-17, and IFN-γ [28,29]. This may provide an alternative for those who are not able to tolerate oral statins at higher doses. 

## 5. Hypothesis

We hypothesise that in rosacea posterior blepharitis, specifically meibomian gland dysfunction, may be a marker for dyslipidaemia and possibly systemic inflammation. Further treatment with statins may not only reduce cardiovascular morbidity and mortality, but also reduce the symptoms and signs of ocular rosacea.

Previous studies have suggested that MGD could be utilised for hypercholesterolaemia screening and that rosacea patients should also be investigated for hypercholesterolaemia [8]. The literature also suggests that MGD-associated cholesterol production dysregulation may be treated locally either through topical or oral statins [24]. There is, however, a gap in the literature with regard to ocular rosacea as yet not considered for the early detection and treatment of local ocular and systemic cholesterol dysregulation and inflammation.

## 6. Testing the Hypothesis

To test this hypothesis firstly with respect to topical statins, a fully funded, ethics-approved, prospective, interventional, blinded pilot study would be planned, for which participants with clinically diagnosed rosacea could possibly be randomised to either receive conventional lid hygiene with placebo, such as topical preservative free lubricants, or conventional lid hygiene with topical Atorvastatin 50 μM, as per Ooi et al. [28]. Treatment would be over an 8-week period. Patients, after enrolment, would be assessed after a washout period at day 0 (Visit 2), week 2 +/− 4 days (Visit 3), week 4 +/− 4 days (Visit 4), and then week 8 +/− 4 days (Visit 5). At each visit, a subjective efficacy evaluation could include the ocular surface disease index (OSDI) dry eye questionnaire and the standard patient evaluation of eye dryness questionnaire (SPEED) [30,31], as well as blepharitis symptom and facial analogue scores, as in our previous pilot study [28]. Additionally, at each visit, objective measures recorded could include tear break-up time, corneal fluorescein staining, and conjunctival injection scores with, for example, the Oculus Keratograph 5M (OCULUS Optikgeräte GmbH, Wetzlar, Germany), as well as the ocular protection index [32], blepharitis sign score [28], and tear film osmolarity measurements, for example, with TearLab Osmolarity System (TearLab, Escondido, CA, USA). Tear sampling for lipidomics with high performance liquid chromatography–mass spectrometry (HPLC-MS) analysis as per Butovich [33] or direct infusion mass spectrometry using electrospray ionisation tandem mass spectrometry (ESI-MS/MS) [34,35] as well as pro-inflammatory cytokines quantification with a multiplex cytokine assay kit could be performed at Visits 2 and 5. The inclusion and/or exclusion of rosacea patients with clinical evidence of Demodex anterior blepharitis could provide (an) additional arm(s) for study.

With respect to oral statin usage, a prospective randomised clinical trial (RCT) to investigate the potential for reduction in blepharitis and hypercholesterolaemia in ocular patients with both MGD and dyslipidaemia could be conducted with high dose versus conventionally dosed oral statins. Similarly, funding would also be required and ethics clearance achieved across multiple interested centres. The statins of choice would be atorvastatin and rosuvastatin, given that they are the most effective at LDL-cholesterol lowering and also the safest in terms of renal function being the least likely to result in new onset microalbuminuria [36]. Evaluation parameters would be much the same as the above topical statin trial but also involve meibography to identify alterations in MG morphology and a longer follow-up. Two initial assessments at 3-month intervals and then at 6 months over the course of a year with a final assessment at 2 years could be an appropriate scheduling, given that RCTs investigating the use of statins in risk reduction in major adverse cardiovascular events have been documented to have a median follow-up of 2.5 years [37]. Secondary outcome measures could include the development of cardiovascular diseases during the study period. Tertiary measures could also include monitoring for the development of and/or amelioration of any concurrent systemic diseases, including diabetes, inflammatory bowel disease, and neuropsychiatric disorders inclusive of depression, as these and others have been significantly associated with rosacea [38].

In the longer-term, prospective studies could be conducted to evaluate the efficacy of oral statins in reducing the risk of developing hypercholesterolaemia in those patients with ocular rosacea who seemingly have cholesterol values within the normal range at the time of treatment. These patients may indeed be present with values already above that which could have been considered healthier for them in terms of their tear film stabilisation as well as cardiovascular status. Prophylactic high-dose rosuvastatin, used for its pleiotropic anti-inflammatory effects in patients with low LDL-cholesterol and elevated high-sensitivity CRP, has been shown to significantly reduce first major cardiovascular events compared to placebo over a median of 1.9 years follow-up [39]. Ocular rosacea patients with low LDL-cholesterol and high CRP levels could be identified and randomised to receive a statin in a similar fashion to observe for the reduction in both MGD and cardiovascular risk.

## 7. Consequences of the Hypothesis and Conclusions

Rosacea is a common inflammatory skin disorder, but its ocular and systemic manifestations are often under-recognised and undertreated. Ocular rosacea-associated MGD may alert clinicians to the presence of and need for the treatment of associated dyslipidaemia and underlying systemic inflammation with statins. If our hypothesis is correct, then the early institution of both topical and oral statins may reduce the burden of ocular rosacea through not only an earlier local ocular surface inflammation control, but indeed a longer-term MG conservation. More importantly, significant cardiovascular morbidity and mortality reduction may follow the earlier introduction of oral statins.

## 8. Patents

The authors have a patent on topical atorvastatin as a novel tear film stabiliser.

## Data Availability

Not applicable.

## References

[1] Albietz J.M. (2000). Prevalence of dry eye subtypes in clinical optometry practice. Optom. Vis. Sci..

[2] Hom M.M., Martinson J.R., Knapp L.L., Paugh J.R. (1990). Prevalence of Meibomian gland dysfunction. Optom. Vis. Sci..

[3] Nelson J.D., Shimazaki J., Benitez-Del-Castillo J.M., Craig J.P., McCulley J.P., Den S., Foulks G.N. (2011). The international workshop on meibomian gland dysfunction: Report of the definition and classification subcommittee. Investig. Opthalmol. Vis. Sci..

[4] Gether L., Overgaard L.K., Egeberg A., Thyssen J.P. (2018). Incidence and prevalence of rosacea: A systematic review and meta-analysis. Br. J. Dermatol..

[5] Marson J.W., Baldwin H.E. (2020). Rosacea: A wholistic review and update from pathogenesis to diagnosis and therapy. Int. J. Dermatol..

[6] Starr P.A., Macdonald A. (1969). Oculocutaneous aspects of rosacea. Proc. R. Soc. Med..

[7] Akpek E.K., Merchant A., Pinar V., Foster C.S. (1997). Ocular rosacea: Patient characteristics and follow-up. Ophthalmology.

[8] Tavassoli S., Wong N., Chan E. (2021). Ocular manifestations of rosacea: A clinical review. Clin. Exp. Ophthalmol..

[9] Ghanem V.C., Neal M., Serena W., Mark M. (2003). The prevalence of ocular signs in acne rosacea: Comparing patients from ophthalmology and dermatology clinics. Cornea.

[10] McCulley J.P., Shine W.E. (2000). Changing concepts in the diagnosis and management of blepharitis. Cornea.

[11] Pinna A., Blasetti F., Zinellu A., Carru C., Solinas G. (2013). Meibomian gland dysfunction and hypercholesterolemia. Ophthalmology.

[12] Duman N., Evans S.E., Atakan N. (2014). Rosacea and cardiovascular risk factors: A case control study. J. Eur. Acad. Dermatol. Venereol..

[13] van Zuuren E.J., Arents B.W.M., van der Linden M.M.D., Vermeulen S., Fedorowicz Z., Tan J. (2021). Rosacea: New Concepts in Classification and Treatment. Am. J. Clin. Dermatol..

[14] Hua T.-C., Chung P.-I., Chen Y.-J., Wu L.-C., Chen Y.-D., Hwang C.-Y., Chu S.-Y., Chen C.-C., Lee D.-D., Chang Y.-T. (2015). Cardiovascular comorbidities in patients with rosacea: A nationwide case-control study from Taiwan. J. Am. Acad. Dermatol..

[15] Palamar M., Degirmenci C., Ertam I., Yagci A. (2015). Evaluation of dry eye and meibomian gland dysfunction with meibography in patients with rosacea. Cornea.

[16] Barbosa E.B., Tavares C.M., da Silva D.F.L., Santos L.S., França A.F.E.D.C., Alves M. (2022). Characterization of meibomian gland dysfunction in patients with rosacea. Arq. Bras. Oftalmol..

[17] Nichols K.K., Foulks G.N., Bron A.J., Glasgow B.J., Dogru M., Tsubota K., Lemp M.A., Sullivan D.A. (2011). The international workshop on meibomian gland dysfunction: Executive summary. Investig. Ophthalmol. Vis. Sci..

[18] Sabeti S., Kheirkhah A., Yin J., Dana R. (2020). Management of meibomian gland dysfunction: A review. Surv. Ophthalmol..

[19] Driver P.J., Lemp M.A. (1996). Meibomian gland dysfunction. Surv. Ophthalmol..

[20] Krenzer K.L., Dana M.R., Ullman M.D., Cermak J.M., Tolls D.B., Evans J.E., Sullivan D.A. (2000). Effect of androgen deficiency on the human meibomian gland and ocular surface. J. Clin. Endocrinol. Metab..

[21] Dao A.H., Spindle J.D., Harp B.A., Jacob A., Chuang A.Z., Yee R.W. (2010). Association of dyslipidemia in moderate to severe meibomian gland dysfunction. Am. J. Ophthalmol..

[22] Braich P.S., Howard M.K., Singh J.S. (2016). Singh, Dyslipidemia and its association with meibomian gland dysfunction. Int. Ophthalmol..

[23] Ooi K.G., Rao A., Goh G.S.-K., Gracie G., Cherepanoff S., Madigan M.C., Watson S.L. (2019). HMG-CoA reductase expression in human eyelid tissue and in a human meibomian gland epithelial cell line. Graefe’s Arch. Clin. Exp. Ophthalmol..

[24] Ooi K.G.-J., Lee M.-H.H., Burlutsky G., Gopinath B., Mitchell P., Watson S. (2019). Association of dyslipidaemia and oral statin use, and dry eye disease symptoms in the Blue Mountains Eye Study. Clin. Exp. Ophthalmol..

[25] Aldaas K.M., Ismail O.M., Hakim J., Van Buren E.D., Lin F.-C., Hardin J.S., Meyer J.J. (2020). Association of Dry Eye Disease With Dyslipidemia and Statin Use. Am. J. Ophthalmol..

[26] Wu K.-I., Chen C.-Y., Jou T.-S., Juang J.-M.J., Lu J.-Y., Wang I.-J. (2020). Effect of 3-Hydroxy-3-Methyl-Glutaryl-Coenzyme A Reductase Inhibitors on the Meibomian Gland Morphology in Patients with Dyslipidemia. Am. J. Ophthalmol..

[27] Yu Y., Maguire M.G., Roy N., Asbell P.A., Yu K., Ying G.-S. (2021). Association of oral statin use with dry eye symptoms and signs in the DRy Eye Assessment and Management (DREAM^©^) Study. Investig. Ophthalmol. Vis. Sci..

[28] Ooi K.G.-J., Wakefield D., Billson F.A., Watson S.L. (2015). Efficacy and Safety of Topical Atorvastatin for the Treatment of Dry Eye Associated with Blepharitis: A Pilot Study. Ophthalmic Res..

[29] Jameel A., Ooi K.G.-J., Jeffs N.R., Galatowicz G., Lightman S.L., Calder V.L. (2013). Statin Modulation of Human T-Cell Proliferation, IL-1β and IL-17 Production, and IFN-γ T Cell Expression: Synergy with Conventional Immunosuppressive Agents. Int. J. Inflam..

[30] Schiffman R.M., Christianson M.D., Jacobsen G., Hirsch J.D., Reis B.L. (2000). Reliability and validity of the Ocular Surface Disease Index. Arch. Ophthalmol..

[31] Ngo W., Situ P., Keir N., Korb D., Blackie C., Simpson T. (2013). Psychometric properties and validation of the Standard Patient Evaluation of Eye Dryness questionnaire. Cornea.

[32] Ousler G.W., Hagberg K.W., Schindelar M., Welch D., Abelson M.B. (2008). The Ocular Protection Index. Cornea.

[33] Butovich I.A. (2013). Tear film lipids. Exp. Eye Res..

[34] Rohit A., Stapleton F., Brown S., Mitchell T., Willcox M. (2014). Comparison of Tear Lipid Profile among Basal, Reflex, and Flush Tear Samples. Optom. Vis. Sci..

[35] Saville J.T., Zhao Z., Willcox M.D.P., Blanksby S.J., Mitchell T.W. (2010). Detection and Quantification of Tear Phospholipids and Cholesterol in Contact Lens Deposits: The Effect of Contact Lens Material and Lens Care Solution. Investig. Opthalmol. Vis. Sci..

[36] Barakat L., Jayyousi A., Bener A., Zuby B., Zirie M. (2013). Comparison of Efficacy and Safety of Rosuvastatin, Atorvastatin and Pravastatin among Dyslipidemic Diabetic Patients. ISRN Pharmacol..

[37] Tramacere I., Boncoraglio G.B., Banzi R., Del Giovane C., Kwag K.H., Squizzato A., Moja L. (2019). Comparison of statins for secondary prevention in patients with ischemic stroke or transient ischemic attack: A systematic review and network meta-analysis. BMC Med..

[38] Haber R., El Gemayel M. (2018). Comorbidities in rosacea: A systematic review and update. J. Am. Acad. Dermatol..

[39] Samson R.H., Nair D.G. (2011). Influence and critique of the JUPITER Trial (Statins v no statins for primary prevention of cardiovascular events in patients with normal lipids and elevated C-reactive protein). Semin. Vasc. Surg..

